# Network ‘Small-World-Ness’: A Quantitative Method for Determining Canonical Network Equivalence

**DOI:** 10.1371/journal.pone.0002051

**Published:** 2008-04-30

**Authors:** Mark D. Humphries, Kevin Gurney

**Affiliations:** Adaptive Behaviour Research Group, Department of Psychology, University of Sheffield, Sheffield, United Kingdom; Indiana University, United States of America

## Abstract

**Background:**

Many technological, biological, social, and information networks fall into the broad class of ‘small-world’ networks: they have tightly interconnected clusters of nodes, and a shortest mean path length that is similar to a matched random graph (same number of nodes and edges). This semi-quantitative definition leads to a categorical distinction (‘small/not-small’) rather than a quantitative, continuous grading of networks, and can lead to uncertainty about a network's small-world status. Moreover, systems described by small-world networks are often studied using an equivalent canonical network model – the Watts-Strogatz (WS) model. However, the process of establishing an equivalent WS model is imprecise and there is a pressing need to discover ways in which this equivalence may be quantified.

**Methodology/Principal Findings:**

We defined a precise measure of ‘small-world-ness’ *S* based on the trade off between high local clustering and short path length. A network is now deemed a ‘small-world’ if *S*>1 - an assertion which may be tested statistically. We then examined the behavior of *S* on a large data-set of real-world systems. We found that all these systems were linked by a linear relationship between their *S* values and the network size *n*. Moreover, we show a method for assigning a unique Watts-Strogatz (WS) model to any real-world network, and show analytically that the WS models associated with our sample of networks also show linearity between *S* and *n*. Linearity between *S* and *n* is not, however, inevitable, and neither is *S* maximal for an arbitrary network of given size. Linearity may, however, be explained by a common limiting growth process.

**Conclusions/Significance:**

We have shown how the notion of a small-world network may be quantified. Several key properties of the metric are described and the use of WS canonical models is placed on a more secure footing.

## Introduction

Networks are widely used to both represent real-world systems for topological study [1] and as a substrate for modeling their dynamics [2]. Many real technological, biological, social, and information networks fall into the broad class of ‘small-world’ networks [3], a middle ground between regular and random networks: they have high local clustering of elements, like regular networks, but also short path lengths between elements, like random networks. Membership of the ‘small-world’ network class also implies that the corresponding systems have dynamic properties different from those of equivalent random or regular networks [3]–[7].

One popular method for studying small-world networks is to use an equivalent network model to generate other similar instances of the class of systems under study. Such generating models may also possess analytic properties that, we assume, may be extrapolated to the target system. One canonical model used as a candidate for network equivalence is the original Watts-Strogatz (WS) model, which has been used as a substrate for studying dynamics in the diverse fields of ecology [8], economics [9], [10], epidemiology [11], [12], and neuroscience [13].

However, the existing ‘small-world’ definition is a categorical one, and breaks the continuum of network topologies into the three classes of regular, random, and small-world networks, with the latter being the broadest. It is unclear to what extent the real-world systems in the small-world class have common network properties and to what specific point in the “middle-ground” (between random and regular) a network generating model must be tuned to genuinely capture the topology of such systems. Here we explore a continuous, quantitative, measure of ‘small-world-ness’, with the aim of overcoming these inadequacies in the current theory of small-world networks.

### Network formalism

When describing a real-world system as a network, each element of the system is represented by a vertex or node, and relationships or interactions between elements are represented by edges between nodes. Two nodes are said to be neighbors if they are connected by an edge, and the *degree k_i_* of node *i* is the number of neighbors it has. The *minimum path length* between two nodes is the minimum number of edges that must be traversed to get from one node to the other. The mean value of the minimum path length over all node pairs will be denoted by *L*.

A key concept in defining small-worlds networks is that of ‘clustering’ which measures the extent to which the neighbors of a node are also interconnected. Watts and Strogatz [3] defined the clustering coefficient 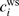 of node *i* by
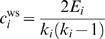
(1)where *E_i_* is the number of edges between the neighbors of *i*. The clustering coefficient of the network *C* ^ws^ is then the mean of 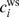 over all nodes. An alternative definition of network clustering in common use [14], based on transitivity, is expressed by
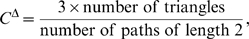
(2)where a ‘triangle’ is a set of three nodes in which each contacts the other two. Both capture intuitive notions of clustering but, though often in good agreement, values for *C* ^ws^ and *C* ^Δ^ can differ by an order of magnitude for some networks. We consider mainly *C* ^Δ^ here, but report where using *C* ^ws^ leads to different results.

A network *G* with *n* nodes and *m* edges is a small-world network [3] if it has a similar path length but greater clustering of nodes than an equivalent Erdös-Rényi (E–R) random graph [15] with the same *m* and *n* (an E–R graph is constructed by uniquely assigning each edge to a node pair with uniform probability). More formally, let *L_g_* be the mean shortest path length of *G* and 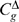 its clustering coefficient using (2). Let *L_rand_* and 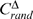 be the corresponding quantities for the corresponding E–R random graph. These ideas may be used to supply a *semi-quantitative* categorical definition of a small world network [3]

#### Definition 1

The network *G* is said to be a small-world network if *L_g_*≥*L_rand_* and 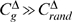.

Here a similar definition applies if we use (1) to define clustering coefficients.

### New measures of small-world-ness

Put
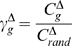
(3)and
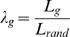
(4)We then define a quantitative metric of ‘small-world-ness’ *S* ^Δ^ according to
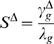
(5)In a similar way, putting
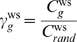
(6)we define *S* ^ws^

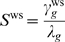
(7)The categorical definition of small-world network above implies λ*_g_*≥1 and 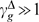, which, in turn, gives *S* ^Δ^>1. We can, therefore, now make a *quantitative* categorical definition of a ‘small-world’ network

#### Definition 2

A network is said to be a small-world network if *S* ^Δ^>1

A similar definition may also be given with respect to *S* ^ws^.

However, notwithstanding the new categorical definition, we wish to emphasize here the utility of using a continuously graded notion of small-world-ness. We go on, therefore, to analyze the properties of the new metrics, and apply them to real-world data for the first time (in [16] we originally proposed this metric as a tool for comparing theoretical neuroanatomy models; its subsequent adoption by others [17], [18], [19] motivated us to consider its theoretical and empirical applications as a universal metric).

## Results

### New metrics behave as required with the Watts-Strogatz model

**Figure 1:**
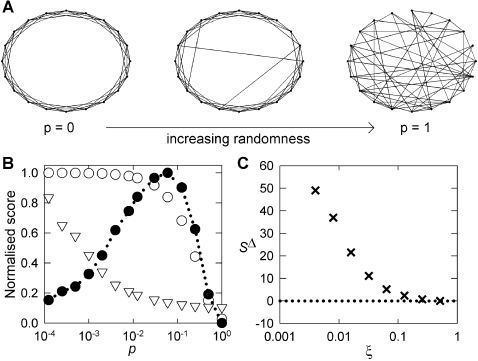
Small-world-ness *S* behaves as required on the Watts-Strogatz (WS) [3] model of small-world networks. A The WS model begins with a ring of *n* nodes, each node connected to its nearest neighbors out to some range *K* (here *K* = 3). Each edge in turn is re-wired to a new target node with probability *p*. B The WS model shows that *p* = 0 gives a regular network, with high clustering but high path length; *p* = 1 gives a pseudo-random network, with low clustering and path length; and intermediate *p* values give small-world networks with high clustering and low path lengths. The *S* metric tracks these changes precisely, and shows which unique *p* value corresponds to high clustering and low path length. Normalized *L* (▿); normalized *C* (○); normalized *S* (•). C The small-world property only applies to sparse networks: densely connected networks trivially have high clustering and short path lengths. With increasing edge density ξ on the WS model, the *S* metric indicates the absence of meaningful small-world structure. A particular edge density for the WS model is obtained by setting *K* = [ξ(*n*−1)/2]. All numerical results obtained on graphs with *n* = 1000, each data-point an average over 20 realizations for each *p* (with *K* = 10) or ξ (with *p* = 0.1) value.

We first checked that the metric *S* ^Δ^ behaves as required on the canonical Watts-Strogatz (WS) model of small-world generation [3]. The WS model begins with a ring of *n* nodes, each node connected to its nearest neighbors out to some range *K*. Each edge in turn is ‘re-wired’ to a new target node with probability *p* (Figure 1A). Values of *p* = 0 and *p* = 1 give regular and random networks, respectively, with intermediate *p* values resulting in ‘small-world’ networks that share properties of both provided that the network is connected and sparse — densely connected networks trivially have small mean path lengths and high clustering coefficients.

Figure 1 shows that small-world-ness captures the topology changes: it has a unique maximum at intermediate values of the re-wiring parameter *p*, indicating the maximum trade-off between high clustering and low path length (Figure 1B), and decays with increasing edge density for a fixed size of network, reflecting the requirement of sparseness (Figure 1C). We can see why this occurs for increasing density. The edge density of a network is given by
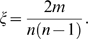
(8)


As ξ→1 then both *C^ws^*, *C* ^Δ^→1 and *L*→1 because all nodes become connected; and as this would apply for both a given real-world network and its E-R random graph equivalent, so *S* ^Δ^, *S* ^ws^→1 regardless of *n*: high edge density results in low small-world-ness.

### Small-world-ness scales linearly with *n* for real networks

**Figure 2:**
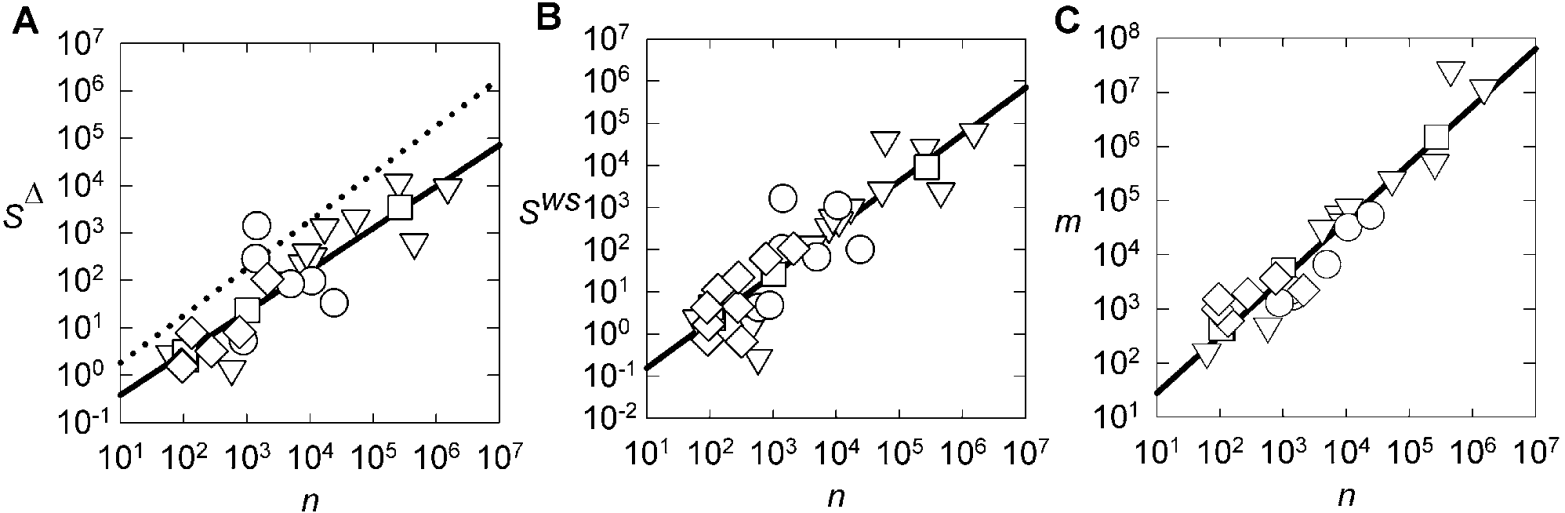
Correlation of real-world network properties. A Small-world-ness *S* ^Δ^ scales linearly with network size *n* across real networks from all domains, and irrespective of their other properties. We show *S* ^Δ^ for all 27 networks for which *C* ^Δ^ could be found or calculated; result was *S* ^Δ^ = 0.023*n* ^0.96^ (*r* ^2^ = 0.78; *p* = 3×10^−9^). The dashed line is the theoretical maximum small-world-ness value of *S* ^Δ^ = 0.181*n* (see text), given the implied mean degree of 〈*k*〉≈5 (see below). B Similarly, using known or calculated *C* ^ws^, we found *S* ^ws^ = 0.012*n* ^1.11^ (30 networks with *S* ^ws^>1; *r* ^2^ = 0.84; *p* = 1.3×10^−11^). C Number of edges *m* also scales linearly with network size *n* — *C* ^Δ^ data-set shown. Best-fit model was *m* = 2.46*n* ^1.06^ (27 networks, *r* ^2^ = 0.92, *p* = 4×10^−15^), implying a mean node degree of 〈*k*〉 = 2*m*/*n*≈5. For the *C* ^ws^ data-set, we found *m* = 3.16*n* ^1.03^ (30 networks, *r* ^2^ = 0.91, *p* = 2×10^−15^) implying 〈*k*〉≈6.32. Residuals of all regressions on log_10_-transformed data did not significantly differ from a normal distribution at *p* = 0.01 (Anderson-Darling test [44]. *C* ^Δ^ data-set, 27 networks: *n* vs *S* ^Δ^: *A* ^2^ = 0.36, *p* = 0.5; *n* vs *m*: *A* ^2^ = 0.45, *p* = 0.28. *C* ^ws^ data-set, 30 networks: *n* vs *S* ^ws^: *A* ^2^ = 0.8, *p* = 0.04; *n* vs *m*: *A* ^2^ = 0.82, *p* = 0.033). Network domains: social (▿); information (); technological (○); biological (⧫).

We computed *S* ^Δ^ and *S* ^ws^ for a broad range of technological, biological, social, and information networks (33 networks in total; Table 1, see Materials and Methods). To our surprise, we found that both forms of small-world-ness scale linearly with the size of the network across all systems falling into the small-world class (Figure 2A,B), irrespective of their originating domain or their other topological properties (e.g. their degree distribution, degree correlation). For *S* ^Δ^, it was not possible to find or calculate *C* ^Δ^ (and hence *S* ^Δ^) for 6 of the 33 networks. However, for the remaining 27, all had *S* ^Δ^>1 and were therefore deemed to be small-world in the new scheme (Definition 2). To ensure the robustness of the categorization, networks with borderline values 1≤*S* ^Δ^≤3 were tested for significance using Monte Carlo sampling of 1000 equivalent E–R random graphs for each network, estimating 99% confidence intervals using standard methods (see Materials and Methods). All such networks had small-world-ness scores significantly greater than an equivalent E–R random graph. For the 27 networks for which *S* ^Δ^>1, linear regression on log-transformed quantities (see Materials and Methods) allowed an estimate of the best power law fit: *S* ^Δ^ = 0.023*n* ^0.96^ (*r* ^2^ = 0.78; *p* = 3×10^−9^). This is an essentially linear scaling of *S* ^Δ^ with *n*.

**Table 1:** Table of small-world-ness values and other topological properties of real networks.

class	#	network	*n*	*m*	〈*k*〉	ξ	*_L_*	*_C_* ^Δ^	*C^ws^*	*S* ^Δ^	*S^ws^*	*p* (WS)	Reference
Social	1	Dolphins†	62	159	5.13	0.084	3.36	0.31	0.26	2.8	2.35	0.64	[41]
	2	film actors	449913	25516482	113.43	_2.5×10_^−4^	3.48	0.2	0.78	627	2446	0.95	[1], [3]
	3	company directors	7673	55392	14.44	0.002	4.6	0.59	0.88	228	341	0.77	[1], [23]
	4	math coauthorship	253339	496489	3.92	_1.6×10_^−5^	7.57	0.15	0.34	11666	26443	0.7	[1], [45]
	5	physics coauthorship	52909	245300	9.27	_1.8×10_^−4^	6.19	0.45	0.56	2026	2521	0.73	[1], [46]
	6	biology coauthorship	1520251	11803064	15.53	_1×10_^−5^	4.92	0.088	0.6	9089	61967	0.88	[1], [46]
	7	email messages	59912	86300	1.44	_4.8×10_^−5^	4.95	-	0.16	-	40524	n/a	[1], [47]
	8	email address books	16881	57029	3.38	_4×10_^−4^	5.22	0.17	0.13	1301	995	0.64	[1], [48]
	9	student relationships	573	477	1.67	0.0029	16.01	0.005	0.001	1.34	0.27	n/a	[1], [20]
	10	newspaper article co-occurence	459	1422	6.2	0.0135	2.98	-	0.02	-	1.67	n/a	[49]
	11	US directors	11057	74414	13.46	0.0012	5.19	0.56	0.87	315	494	0.77	[50]
	12	UK directors	8850	39741	8.98	0.001	6.46	0.61	0.89	386	561	0.71	[50]
	13	German directors	4185	30438	14.55	0.0035	6.4	0.72	0.93	100.71	129.7	0.79	[50]
Information	14	WWW nd.edu	269504	1497135	5.56	_4×10_^−5^	11.27	0.11	0.29	3453	9104	0.81	[1], [51]
	15	Roget's Thesaurus	1022	5103	4.99	0.0098	4.87	0.13	0.15	23.54	27.17	0.76	[1], [52]
	16	word adjacency†	112	425	7.59	0.0684	2.54	0.16	0.17	2.13	2.34	0.74	[26]
	17	book purchases†	105	441	8.4	0.081	3.08	0.35	0.49	3.09	4.33	0.71	V.Kreb, unpublished (www.orgnet.com)
Technological	18	Internet	10697	31992	5.98	_5.6×10_^−4^	3.31	0.035	0.39	98.09	1093	0.83	[1], [53]
	19	power grid	4941	6594	2.67	_5.4×10_^−4^	18.99	0.1	0.08	84.45	67.56	0.8	[1], [3]
	20	train routes	587	19603	66.79	0.114	2.16	-	0.69	-	4.26	n/a	[1], [54]
	21	software packages	1439	1723	1.2	0.0017	2.42	0.07	0.082	1403	1644	n/a	[1], [25]
	22	software classes	1377	2213	1.61	0.0023	1.51	0.033	0.012	285.26	103.73	n/a	[1], [55]
	23	electronic circuits	24097	53248	4.42	_1.8×10_^−4^	11.05	0.01	0.03	33.5	100.5	0.91	[1], [56]
	24	peer-to-peer network	880	1296	2.95	0.0034	4.28	0.012	0.011	5.26	4.82	0.85	[1], [57]
Biological	25	metabolic network	765	3686	9.65	0.0126	2.56	0.09	0.67	8.18	60.89	0.82	[1], [58]
	26	yeast protein interactions	2115	2240	0.001	2.12	6.8	0.072	0.071	107.85	106.35	0.73	[1], [59]
	27	marine food web	135	598	4.43	0.0661	2.05	0.16	0.23	7.84	11.27	0.64	[1], [60]
	28	freshwater food web	92	997	10.84	0.2382	1.9	0.2	0.087	1.7	0.74	0.74	[1], [21]
	29	C.Elegans†	277	1918	13.85	0.05	2.64	0.2	0.28	3.21	4.51	0.81	[42]
	30	Macaque cortex†	95	1522	32.04	0.34	1.78	0.7	0.77	1.53	1.69	0.79	[42]
	31	E. Coli substrate	282	1036	7.35	0.0261	2.9	-	0.59	-	22.08	n/a	[22]
	32	E. Coli reaction	315	8915	56.6	0.18	2.62	-	0.22	-	0.67	n/a	[22]
	33	functional cortical connectivity	90	405	9	0.1	2.49	-	0.53	-	4.32	n/a	[17]

Entries ‘-’ indicate missing data; n/a indicates values that could not be computed. All *S* ^Δ^,*S* ^ws^, edge density ξ and implied *p*(WS) were computed by us; for networks marked ^†^ we have computed some or all of 〈*k*〉, *L*, *C* ^Δ^ and *C* ^ws^ from available data-sets. References are given for the source of the original network data, and also for the analyses where these were done separately.

For *S* ^ws^, 3 networks in our data-set were not small-worlds: relationships amongst students [20] *S* ^ws^ = 0.27 (network #9); a freshwater food web [21], [1] *S* ^ws^ = 0.74 (network #28); and the E-Coli reaction graph [22] *S* ^ws^ = 0.67 (network #32). This demonstrates both that the small-world property is not robustly achieved for small networks, and that it is contingent on the particular measure of clustering used. Once again, networks with borderline values 1≤*S* ^ws^≤3 were tested using Monte Carlo methods for significant membership of the small-world category and were found to satisfy this criterion. For the 30 networks with *S* ^ws^>1, a similar regression to that used for *S* ^Δ^ gave *S* ^ws^ = 0.012*n* ^1.11^ (*r* ^2^ = 0.84; *p* = 1.3×10^−11^). Thus, there is also a robust linear scaling of *S* ^ws^ with *n* (see Text S1 for further details).

### Linear scaling on the Watts-Strogatz model

Linear scaling of small-world-ness with network size was unexpectedly shown by the canonical Watts-Strogatz model [3] of small-world network generation. We now show that this result can be explained analytically. In what follows, many of the relationships are held only approximately, but because these approximations are often very good we show them as equalities. Note that from here on we use subscript names to identify analytic quantities that pertain to a particular network model only.

If *L_ws_* is the mean shortest path length in the WS model, then it is known [14] that
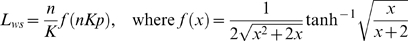
(9)Similarly, it is known for E-R random graphs [23] that
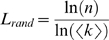
(10)where 〈*k*〉 is the expected value of the degree across the network. In the WS model, node degree and range are related by 〈*k*〉 = 2*K*, so that
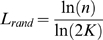
(11)Using (11), (9) and (4) the path length ratio λ*_ws_* for the WS model is
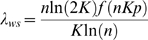
(12)The function *f*(*x*) in (9) has an upper asymptote of ln(2*x*)/4*x* if 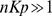. Thus, assuming 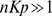, (12) becomes
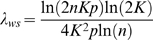
(13)If 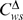 is the clustering coefficient of the WS model (using (2) as the metric), then it is known [24] that
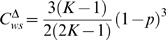
(14)For E–R random graphs [23], to a good approximation, 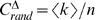, so that using 〈*k*〉 = 2*K*

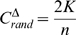
(15)Therefore, using, (14), (15) and (3)
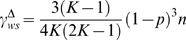
(16)From (5), (16) and (13),
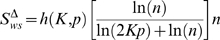
(17)where *h*(*K*, *p*) is a function of *K* and *p* only. The term in the square brackets tends to 1 as *n*→∞ and so, for large enough *n*, *S* ^Δ^ for the WS model scales with *n*. To quantify this approximation, we performed a linear regression on log-transformed quantities (just as for the real networks) over the typical range of *n* encountered in our sample of networks, 10^2^≤*n*≤10^7^, and found a linear fit, with *r* ^2^ within 10^−5^ of unity.

### Establishing the precise WS model correlate of a real network

The WS network is often used as a generative model for real small-world networks [e.g. 8]–[13]. This is assumed to establish a ‘first-pass’ model of that system’s topology, which may be augmented by considering other factors such as degree sequence [23], degree correlation [25], modularity [26] and other properties.

In matching the WS parameters *K*, *p*, *n* to the target system, we know *n*, can measure 〈*k*〉 (giving *K* = 〈*k*〉/2), but estimating *p* has, hitherto, remained problematic. However, using our new metric of small-world-ness, it is possible to establish *p* in a principled way. Thus, if *G* is a real (target) network with measured small-world-ness 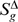, we identify it with the WS network with the same value of *S* ^Δ^. That is, form 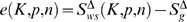, where 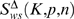 is given by the right hand side of (17), and minimise *e* with respect to *p*, keeping *K*, *n* at their measured values. We did this for our sample of real-world systems, omitting those for which 〈*k*〉≤2 since the expressions used in defining 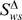 are inaccurate in these cases (we used Matlab routine fzero, initial value of *p* = 0.5). The resulting *p* values for the equivalent WS model are listed in Table 1

Given that the real-world networks showed *S* ^Δ^∝*n*, the WS networks derived from them under the procedure described here must do likewise (they have identical *S* ^Δ^ values). However, the result in the previous section would suggest that this implies *K*, *p* are roughly constant for this set of WS networks.

To investigate the constancy of *K* we used the result that 〈*k*〉 = 2*m*/*n* (where *m* is the number of edges in the network). So, using *K* = 〈*k*〉/2, constant *K* is equivalent to establishing *m*∝*n*. Figure 2C shows the result of regressing *m* against *n* (using log-transformed quantities) for the real world networks. For networks with *S* ^Δ^>1, the best fit model was *m* = 2.46*n* ^1.06^ (27 networks, *r* ^2^ = 0.92, *p* = 4×10^−15^), implying a mean node degree of 〈*k*〉 = 2*m*/*n*≈5; for networks with *S* ^ws^>1, the best fit model was *m* = 3.16*n* ^1.03^ (30 networks, *r* ^2^ = 0.91, *p* = 2×10^−15^) implying 〈*k*〉≈6.32. Thus, the real-world networks fulfill the prediction of constant mean node degree. A similar result holds for *p* values; we found that all testable real-world systems fall into a very limited range of *p* for the equivalent WS model (0.64≤*p*≤0.95 and σ*_p_* = 0.0806).

An alternative view of these results is as follows. We could start with the empirically observed approximate constancy of mean node degree 〈*k*〉 and calculated rewiring parameter *p* for the real world networks, and deduce a linear scaling of *S* ^Δ^ for the WS models. Then, under the equivalence of *S* ^Δ^ for both real-world networks and their WS counterparts, we could have predicted that *S* ^Δ^ for the real-world networks would also scale linearly.

### The linear scaling of small-world-ness with *n* is not inevitable

Is the relationship *S*∝*n* inevitable for all systems? (The subsequent argument holds for *S* based on either definition of clustering coefficient and so superscripts Δ, ws are dropped). To investigate this we note that it is always possible to write *S_i_* = α*_i_n_i_* for the *i*th system, for some value α*_i_*; in the case of linear scaling, α*_i_* is constant. To proceed further, we now express α*_i_* in terms of other system parameters. Using the definition of *S* and (10) for random graphs,
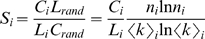
(18)where *C_i_*, *L_i_*, 〈*k*〉*_i_* are the clustering coefficient, mean shortest path length, and mean node degree of system *i* respectively. While we do not know exactly how *L_i_* depends on *n*, we note that the mean shortest path length for small-world networks is usually assumed to scale logarithmically like random graphs: from (11), *L_rand_* = [1/ln(〈*k*〉)]ln(*n*); and for the WS model, using (9) with large *n*, *L_ws_* = (1/4*K* ^2^ *p*)ln(*n*). Both relations are of the form *L* = βln(*n*) where β is independent of *n*. We therefore write *L_i_* = β*_i_*ln(*n_i_*), where β*_i_* is the factor that ensures the equality to be true (i.e it plays a similar role in this respect as α*_i_*).

This gives
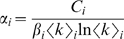
(19)In general, there is no *a priori* reason to suppose that the variables *C_i_*, 〈*k*〉*_i_* and β*_i_* are either all constant, or co-vary in a way commensurate with constancy for α*_i_*. However, for the sample of networks used here, as noted above, the mean node degree 〈*k*〉*_i_* is approximately constant. It is now instructive to see how much co-variation is required between the remaining two variables in order to ensure a significantly different power law holds between *S* and *n*.

Thus, suppose that we fit a model *S* = μ*n* ^1.5^ so that we expect 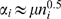. For the range of *n* encountered here – approximately four orders of magnitude – α*_i_* would therefore have to range over 2 orders of magnitude. For this to occur, there must be sufficient variation in *C_i_* and β*_i_*, and these two quantities should correlate well with *n*. The ranges of the two variables are reasonably large in the data-set – using *C* ^Δ^, 0.209≤β*_i_*≤2.52 and 0.005≤*C_i_*≤0.72 – and could plausibly generate the required 100-fold variation. However, the correlation coefficients with *n* are very small: for β*_i_*, *r* ^2^ = 0.028 and for *C_i_*, *r* ^2^ = 0.025. This would therefore appear to preclude a nonlinear relationship between *S* ^Δ^ and *n* for the networks studied here.

To study the effect of a lack of correlation between *n* and network parameters like *C_i_* on linear scaling between *S* ^Δ^ and *n*, we ran a Monte Carlo simulation (see Materials and Methods). Each one of 1000 experiments consisted of sampling 27 randomly drawn *C* values for networks with constant β, and with a spread of *n* over 4 orders of magnitude. For each network its small-world-ness was computed and a linear regression of *S* against *n* performed. This resulted in a mean of *r* ^2^ = 0.89±0.06 s.d. across the 1000 experiments, showing the strong tendency for linearity in this case.

**Figure 3:**
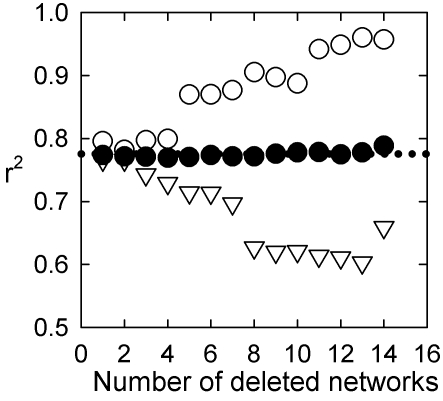
Robustness of WS model prediction *S*∝*n.* We test the effect of real-world networks deviating from the constant 〈*k*〉 assumption using the following iterative procedure: (i) regress *n* vs *m* for the data-set (as in Figure 2C); (ii) select network to remove from data-set based on regression outcome; (iii) regress *n* vs *S* for reduced data-set and record new goodness-of-fit (as *r* ^2^); (iv) repeat from (i) until 50% of networks removed. We do this for 3 selection cases, based on *S* ^Δ^ here, in step (ii): (a) removing the network with the largest deviation from *m*∝*n* linear model increased the goodness-of-fit (○) for the *S*∝*n* linear model (the dotted line indicates the original goodness-of-fit value at *r* ^2^ = 0.78); (b) removing networks with the smallest deviation from *m*∝*n* linear model decreased the goodness-of-fit (▿) for the *S*∝*n* linear model; (c) random deletion did not consistently change the goodness-of-fit (•). Thus deviation from the assumption of constant 〈*k*〉 correlates with deviation from the linear *S*∝*n* model for the real-world networks, as predicted by the WS model. In addition, case (c) shows that the linear *S*∝*n* model is robust to taking random sub-sets of the networks. Identical trends were obtained for *S* ^ws^. All random deletion data-points averaged over 1000 realizations of the regress-delete-regress sequence; both largest and smallest deviation cases were unique sequences.

The linear relationship is, however, sensitive to deviations from the approximation that 〈*k*〉 is constant. That is, networks that deviate furthest from the linear *m*∝*n* model in turn deviate furthest from the linear *S*∝*n* model (Figure 3). This was shown using a novel regress-delete-regress procedure outlined in Materials and Methods (we were able to directly test the sensitivity to 〈*k*〉, rather than using a Monte Carlo approach as above, because the strong correlation of *m* with *n* provided a baseline from which we could quantify deviation of 〈*k*〉 from constancy). Further, Figure 3 shows that if we delete a random set of networks from the data-set, then the average effect is to not change the fit to the linear *S*∝*n* model: the linear scaling is robust, and does not depend on a specific network set.

The sensitivity of small-world-ness linearity with *n* to degree 〈*k*〉 suggests that introduction of networks with very high edge density into our sample would destroy the linear scaling. We can rewrite (8) using 〈*k*〉 = 2*m*/*n*

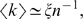
(20)and see that mean degree scales linearly with edge density. Thus, a network with high edge density implies high mean degree, which in turn would fall far from the linear *S* = α*n* model, as we have just shown.

One exemplar of a real system with high edge density is the network of individual neurons within a single vertebrate brain region. Detailed network data for these are not available because of the great technical difficulties in reliably reconstructing even small networks such as the 302 neuron *C. Elegans* nervous system [27]. Indeed, high edge density itself may be the primary cause of technical problems in reconstructing complete systems from many domains, resulting in their absence from the network literature. Nonetheless, approximate reconstructions can be attempted. Quantitative anatomical models of individual brain regions suggest that each of the hundreds of thousands or millions of neurons receive many thousands of connections, and each themselves connect to similar numbers of target neurons [16], [28]. Such networks of neurons can have very low small-world-ness values for their size [16], and thus fall far from the linear *S*∝*n* model discovered here.

We conclude here that the linear relationship between small-world-ness and system size does not hold for an arbitrary collection of networks, but is highly likely if all such networks have a similar mean node degree.

### Other scaling properties of small-world-ness

Having established that *S* scales linearly with *n*, it is also instructive to look at how its component ratios scale with *n*. We find, as expected, that most networks falling into the small-world class have approximately the same mean shortest path length as their equivalent E–R random graphs, and so λ≈1. Given this, it is unsurprising that both γ^Δ^ and γ^ws^ then scale linearly with *n* (see Figure S1). We did find that three networks in our data-set — email messages (#7), software packages (#21), and software classes (#22) — had λ≈0.1, indicating that their mean shortest path length was an order of magnitude smaller than the equivalent E–R random graph. These networks are thus *ultra-small* [29], and indeed both email message (#7) and software package (#21) networks fall further from the linear model than any others.

Given the existence of the linear scaling with *n*, the scaling of small-world-ness with some other topological properties is completely determined. We can directly determine from (20) how edge density behaves in our data-set (values for ξ are given in Table 1). Taking our fitted linear model *S* = α*n*, we can substitute *n* = *S*/α in (20) and find that
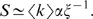
(21)Substituting our found values of 〈*k*〉 and α for the fits to either *S^ws^* or *S* ^Δ^ confirms that this is a good approximation. Therefore, because small-world-ness linearly scales with network size, and degree is approximately constant, then *S* also has a simple inverse linear scaling with edge density.

### Real-world systems do not maximize small-world-ness

We can show that the specific scaling coefficient α in the relationship *S* = α*n* for the real-world networks studied here does not maximize small-world-ness for a particular size of network. First, we show that the WS model predicts an approximately constant amount of rewiring *p* that maximizes *S* ^Δ^, independent of network size. To do this, given the above analytic expressions (13) for λ*_ws_* and (16) for 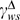 (and again assuming 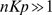), we found 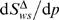, and set 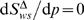. Solving this equality for *p* would then give us the value of *p* that maximized 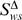, if one existed — see Text S1 for details of the solution.

We did this over the range 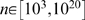 with *K* = 〈*k*〉/2 = 2.5, since this is implied by the result of Figure 2C. If *p* ^*^ is the value of *p* giving maximal 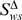, we found that for small networks (*n* = 10^3^) *p* ^*^ = 0.222 and, as *n*→∞, *p* ^*^→0.246, so that the range of *p* ^*^ is very small. The constant *K* and very small range of *p* ^*^ imply that the associated maximum 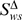 values should scale linearly with *n*. It transpires that the theoretical maximum 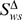 depends almost exactly in a linear way on *n* with slope 0.181 (and plotted in Figure 2A). Thus, *S* ^Δ^ is not maximized by the real-world networks.

### A generative mechanism for a specific linear *S*∝*n* relationship

We have established and explained many simple properties of real-world networks and of their equivalence class in the WS model. We now show how the specific, sub-maximal, linear scaling of *S* = α*n* could have been generated. The models we examine here are intended as informative examples of the generation and limits on *S* scaling, not an exhaustive list of those which could generate the specific linear scaling we found — that remains the subject of future work.

Many of the real-world systems share common generative principals despite their widely differing origins. Most systems have a growth process, showing some form of preferential attachment [30] that is limited by the cost of adding new edges and by the capacity to maintain them (as might be induced by aging) [31]. Simple models of this process result in ‘scale-free’ networks with power-law or truncated power-law degree distributions [30], [31], a property that is also common to many real-world systems considered here [32] (but see [33] for an alternative view of some biological networks). However, networks generated by these models are not ‘small-world’ by either Definition 1 or 2. Their clustering coefficient is inversely proportional to *n*, going to zero as *n* grows large [34]. Thus, they cannot show linear scaling of *S*: it is at best constant and at worst goes to zero with increasing *n*.

A noisy, limited growth process *can* generate the specific linear *S* = α*n* relationships we report. A generalized form of the Klemm-Eguiluz model (GKE)[34], [35] encapsulates this process, and has the unique property of creating networks that are both small-world (short path length, high clustering) and ‘scale-free’ (having a truncated power-law degree distribution) as found for many real-world systems considered here. (To the best of our knowledge, all known real-world systems with power-law-like degree distributions also fall into the broad ‘small-world’ class we discussed in the Introduction; it is only the scale-free networks formed by the simple models that form a distinct set of ‘scale-free-only’ networks). By using the GKE model, we therefore also show that linear scaling of *S* can occur whether or not the real-world systems have ‘scale-free’ properties.

The GKE model begins with an active set of *M* nodes. At every time-step a new node is added, connecting *d* edges: one edge added to a random inactive node with probability ρ, adding noise to the process; all remaining edges connect to randomly chosen active nodes. One of the active nodes is deactivated with a probability proportional to the active nodes’ degrees; finally, the new node is activated. The sequence repeats until the desired size of network is obtained.

**Figure 4:**
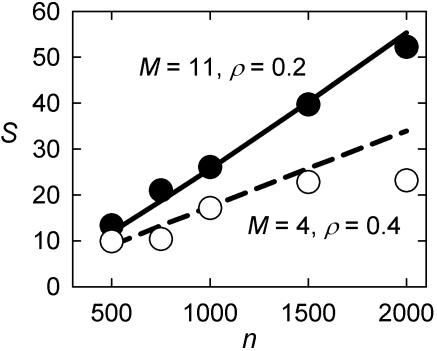
A limited growth process can generate the observed *S*∝*n* relationships. We created 5 networks for every value of 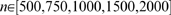 using a generalized form of the Klemm-Eguiluz model (GKE) [34], [35], setting *d* = 3 so that the resulting networks had the same mean degree (∼6) as we found for the real-world systems. We searched on the GKE model's *M* and ρ parameters (see text) to minimize the root mean square error between the resulting scaling of the averaged *S* ^ws^ (•) and *S* ^Δ^ (○) values and the observed real-world network relationships (respectively, the solid and broken lines). The best-fit parameters were different for the two forms of *S*, underlining that they measure two different properties of the real systems.

We found that specific values for *M* and ρ could generate GKE networks with the same linear scaling relationships between network size and *S* ^ws^ and *S* ^Δ^ that we observed for the real-world networks (Figure 4; see Materials and Methods, and Figure S2). Therefore, a possible general mechanism for particular linear scaling rates of small-world-ness is a common size of both active node set and quantity of noise during creation of the real-world systems.

## Discussion

Small-world-ness is a topological property linking real-world systems across domains of research. Hitherto it has been defined only in semi-quantitative way (Definition 1). In this paper we propose quantitative measures of small-world-ness – *S* ^Δ^ and *S* ^ws^ – and define a network to be in the small-world category with respect to either of them if the small-world-ness is greater than 1 (e.g Definition 2). This quantification of small-world-ness allows for the statistical testing of its presence in any given network.

The Watts-Strogatz (WS) model plays a key role in the study of small-world networks. It uses a generative process to create classes of small-world network and is now widely used as a model for studying dynamic systems [3]–[13]. However, until now, a precise parameterization of the WS model associated with a given kind of real-world network remained elusive. Our introduction of a quantitative measure of small-world-ness remedies this by demanding that the WS counterpart to a specific network have the same value of *S* ^Δ^ (or *S* ^ws^). For the WS models it is possible to show analytically that, under certain circumstances (constant re-wiring parameter and range), the small-world-ness *S* ^Δ^ will scale linearly with network size *n*. Intriguingly, a wide class of real-world networks also shows this linear scaling. Given this similarity in behavior, the assumption of (limited) topological correspondence of the WS model with real networks implies certain constraints on empirically measured parameters (like mean degree) of these networks. These constraints appear to hold, and so the ideas developed here provide further support for using the WS model in the study of small-world systems.

We have shown that the linear scaling between *S* ^Δ^ and *n* is not an inevitable property of networks; it would be possible, for example, to include networks with very large edge density that would destroy any linear scaling. However, in the event of linear scaling, there is a variety of possible scaling constants and there is a noisy growth process that could give rise to the networks sharing the same scale (slope) parameter. Finally, we have shown that the small-world networks used here do not maximize *S* ^Δ^ (there are ‘steeper’ linear relationships between *S* ^Δ^ and *n*).

We cannot, on the basis of the work presented here, answer the question of why small-world-ness was not maximised, but we can give some insights as to why this is the case. The possible explanations split into two broad classes of structural and dynamical limitations. Our use of the GKE model showed that the limited capacity of a system's nodes to maintain edges (whether due to physical cost, aging processes, or some other mechanism) is one structural limitation that could result in sub-maximal small-world-ness. Other structural limitations could include physical limits on node location and length of edges, such as might occur for the sub-stations and transmission wires in the power grid network.

Even if structural limitations were not an issue, then the system may have dynamical requirements that prevent it from maximising small-world-ness. The constraints placed on a system's topology by the dynamics required to fulfill its function are not well understood. Recent work has shown how the presence of particular network ‘motifs’ — repeating patterns of connections between a small number of nodes — can guarantee, for example, a chaotic attractor for the network as a whole [36]. The functional requirements of some real-world system may then lead to the inclusion of particular motifs to guarantee the necessary dynamics [37], and there is no necessary link between a system's motifs and its global topological properties (of which small-world-ness is but one). Nonetheless, given that so many of the key motifs identified so far are either complete 3-node loops or contain them [37], [38], the global topology will have a high clustering coefficient, and will most likely be a small-world network.

Other systems may have constraints placed directly on their global topology, and this too could prevent maximisation of small-world-ness. For example, in his original work on the small-world model, Watts [39] explored the dynamics of Kuramoto oscillators on a WS model substrate, and showed that the fraction of synchronised oscillators had a phase transition that occurred for progressively smaller *p* as the oscillators' symmetric coupling strength increased (for fixed *n*, *K*). Therefore, if a system's function required it to be at the phase transition, so that it could rapidly switch between synchronised and desynchronised states with minimal perturbation, the required amount of (implied) rewiring may be far from that which maximised small-world-ness.

These are just a few of many possible explanations for why real-world systems do not maximise small-world-ness. Instead we might ask, when would small-world-ness be maximised? Maximum *S* essentially identifies the point in the network's possible topologies where the highest clustering is achieved for the smallest deviation from the shortest mean path length. Such a network would be optimal for message-passing, such that all the nodes receive a message in the shortest possible number of network steps [40]. On this basis, we expect that some form of dynamic phenomenon, whether based on percolation (or, equivalently, epidemiological SIR models), oscillators, or some other general ordinary differential equation system, will have a strong correlation with small-world-ness. So, just as we have used a continuously graded ‘small-world-ness’ to quantitatively examine the topologies of the broad class of small-world networks, we may use this as the starting point for quantifying the continuum of dynamic properties that must also span this class.

## Materials and Methods

### Data-set of real-world systems

We collated a database of real-world networks' topological properties, combining published results with our own analyses of available data-sets. These are presented in Table 1, extending the previous considerable effort of collating topological properties by Mark Newman [1]. All networks are treated as undirected. We list 33 real-world systems in total: we could compute *S* ^ws^ for all systems and *S* ^Δ^ for 27 systems — *C* ^Δ^ could not be found or computed for those systems.

We emphasise that the networks were not chosen for their ability to fit the linear model of *S*∝*n*. The majority of the data-set (21 of 33) were obtained from a previous collation [1]: networks were only omitted from that prior data-set if neither *C* ^ws^ or *C* ^Δ^ were available for them (and hence were of no practical use to us here). Many of the additional networks we added filled sub-domains missing from the prior data-set, for example: the dolphin network [41] is an example of an animal social network; the cortical area connectivity map [42] is an example of large-scale neural connectivity. In addition, the regress-delete-regress sequence we used in the main text (and see below) shows two properties. First, that we could have applied that method to the data-set in Table 1 before further analysis, pruning the data-set down to those networks that showed the best fit to the linear model (by choosing the ‘most-deviant’ networks to omit), but did not. Second, that the linear scaling property is robust across randomly chosen sub-sets of the network data-set: on average, randomly deleting networks from the data-set did not significantly reduce the fit to the linear model.

### Testing significance of *S* scores

We assess the significance of borderline small-world-ness scores *S*1 using Monte Carlo methods. The null hypothesis for the Watts-Strogatz definition of small-world networks is that the system is an Erdös-Rényi (E–R) random graph. We thus constructed *N* = 1000 E–R networks with the same number of nodes *n* and edges *m* for each tested real-world system, computing 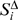 and 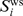 for the *i*th E–R network. The 99% confidence limits for the null hypothesis were then defined for each system. We first found the central 99% interval [a*, b*], that is [43]

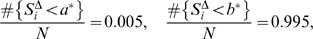
(22)and similarly for *S* ^ws^. The 99% confidence interval for the system is then
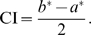
(23)The upper 99% confidence limit is then CL^0.01^  =  1 + CI (where by definition *S* ^ws^, *S* ^Δ^ = 1 for an E–R random graph). A network with *S*>CL^0.01^ was therefore considered to significantly differ from a random network. We note that adopting a quantitative definition (Definition 2) of small-world-ness has led us to a procedure for a general statistical test for the presence of small-world structure, as defined by Watts and Strogatz [3], which is particularly useful for establishing meaningful departures from randomness in small networks.

### Fits to linear scaling

Least-squares regressions on small-world-ness *S* and number of edges *m* against size of system *n* were performed on log_10_-transformed data to normalize magnitude of errors across range of *n*. Best fit linear model log_10_(*x*) = *a*+*b*log_10_(*n*) back-transformed to a linear basis, giving *x* = α*n* ^β^, where α = 10*^a^* and β = *b*. MATLAB (Mathworks) function **regress** was used to perform the regressions. The validity of *r* ^2^ significance values was established by confirming that the residuals of each regression had a normal distribution at *p* = 0.01 using the Anderson-Darling test [44].

### A regress-delete-regress procedure for testing robustness of predictions

We test the effect of real-world networks deviating from the constant 〈*k*〉 assumption using the following iterative procedure:

1. regress *n* vs *m* for the data-set (as in Figure 2B);
2. select network to remove from data-set based on regression outcome (3 different selection criteria were used, detailed below);
3. regress *n* vs *S* for reduced data-set and record new goodness-of-fit (as *r* ^2^);
4. repeat from step 1 until 50% of networks removed.

We do this for 3 selection cases in step 2. First, we tested removing the network with the largest deviation from *m*∝*n* linear model in each iteration, hypothesizing that this should lead to an overall increase in fit to a linear model (increased *r* ^2^) for *S* = *f*(*n*) if the WS model behaviour reflected that of real-world systems. Second, we tested removing the network with the smallest deviation from *m*∝*n* linear model at each iteration, hypothesizing that this should lead to an overall decrease in fit to a linear model (decreased *r* ^2^) for *S* = *f*(*n*) if the WS model behaviour reflected that of real-world systems. Third, we tested random deletion, where a random network was deleted at each iteration, irrespective of the regression outcome, to establish the baseline effect of removing systems from the data-set. The first and second cases are unique sequences of deleted networks; the third case we repeated 1000 times.

### Monte Carlo testing of linear scaling

We tested the dominance of linear *S*∝*n* scaling given an approximately constant mean node degree 〈*k*〉 and path length scaling β. For each Monte Carlo simulation, we drew 27 random *C* values from a uniform distribution in [0,1], computing α*_i_* for each from Eq. (19) with constant β*_i_* = 1 and 〈*k*〉*_i_* = 6. We then computed *S_i_* = α*_i_n_i_* for each, using a logarithmic spread of 27 network sizes 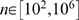 to closely match the spread of the real-world system sizes. Linear regression (as detailed above, including the log_10_-transform) was then performed on the set of simulated 27 *S* values and the *r* ^2^ recorded. We repeated this procedure 1000 times.

### Searching GKE model parameter space

We wished to determine if the generalized Klemm-Eguiluz model (GKE)[31], [32] model could explain the particular scaling relationships we found for the real-world systems:
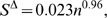
(24)

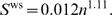
(25)


We explored the (*M*, ρ) parameter space, searching over 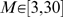 in steps of 1, and 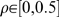 in steps of 0.1. The lower limit on *M* is set by the number of edges added per new vertex, and here we set *d* = 3 to give a mean degree of 〈*k*〉≈6 for a GKE model network, approximately the same degree that was implied by the linear *m*∝*n* relationship for the real-world systems. For each (*M*, ρ) pair, we constructed 5 GKE model networks for each value of 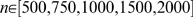 and computed their *S* ^ws^ and *S* ^Δ^ scores. We took the mean of these 5 scores for each *n*, giving sets 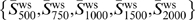 and 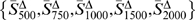. The fit of the GKE model networks was then assessed by computing the root mean square error (RMSE) between these mean values and those given by the scaling relationships (24) and (25) for the tested sizes of network *n*. The parameters that minimised RMSE are given in Figure 4; the error landscapes are shown in Figure S2.

## Supporting Information

Text S1 Supporting information text
(0.22 MB PDF)

Figure S1 Correlation of real-world systems' clustering coefficient and path length ratios with system size. Clustering coefficient ratios (a) γ^Δ^ = *C* ^Δ^ *C*_rand_ and (b) γ^ws^ = *C* ^ws^ *C*_rand_ both scaled linearly with *S*. Linear regressions found *r* ^2^≅0.86 in both cases. (c) As expected for small-world-networks, path length *L* was approximately the same as that of an E-R random graph, and so λ = *L*/*L*_rand_1 for most networks (note that we show λ here for all 33 networks). All linear regressions performed on log_10_-transformed data, as detailed in Materials and Methods of the main text.
(0.11 MB TIF)

Figure S2 The root mean square error (RMSE) distribution across tested values of the GKE model parameters. The RMSE is computed based on the difference between the mean values of small-world-ness for a set of generated GKE networks and the corresponding small-world-ness values from the specific linear relationships found for the real-world systems. (a) RMSE error distribution for the fit to the *S* ^ws^∝*n* relationship. RMSE plotted on log scale to emphasise valley of minimum values. Stick-and-ball indicates the parameter pair that minimised RMSE. (b) RMSE error distribution for the fit to the *S* ^Δ^∝*n* relationship. Stick-and-ball indicates the parameter pair that minimised RMSE.
(0.44 MB TIF)
