# Supplementary material for: Network ‘Small-World-Ness’: A Quantitative Method for Determining Canonical Network Equivalence
Source: PLoS One. 2008 Apr 30;3(4):e2051. doi: 10.1371/journal.pone.0002051 (PMC2323569; doi:10.1371/journal.pone.0002051)
Supplement: Text S1 — Supporting information text (0.22 MB PDF) [file pone.0002051.s001.pdf]

Supporting Information

Network 'small-world-ness': a quantitative method for determining canonical network equivalences

Mark D. Humphries and Kevin Gurney

Adaptive Behaviour Research Group,
Department of Psychology, University of Sheffield,
Sheffield. S10 2TP. UK
m.d.humphries@sheffield.ac.uk

1 Real-world systems — details

The correlations of $S^{\triangle}$ and S^{ws} with n (see main text) are re-plotted in Figure ST1a and Figure ST1b, respectively, using the indices of the networks from Table 1. The three real-world systems with $S^{\text{ws}} < 1$ were omitted from that correlation as these were not small-world networks. Some systems were borderline small-world networks, defined here as $1 \leq S^{\triangle}, S^{\text{ws}} \leq 3$ — 4 systems had S^{ws} in this range, 6 systems had $S^{\triangle}$ in this range. For these we tested the significance of their small-world-ness scores as detailed in the main text. All had S values greater than the upper 99% confidence limit. We conclude that all other real-world systems were small-world networks.

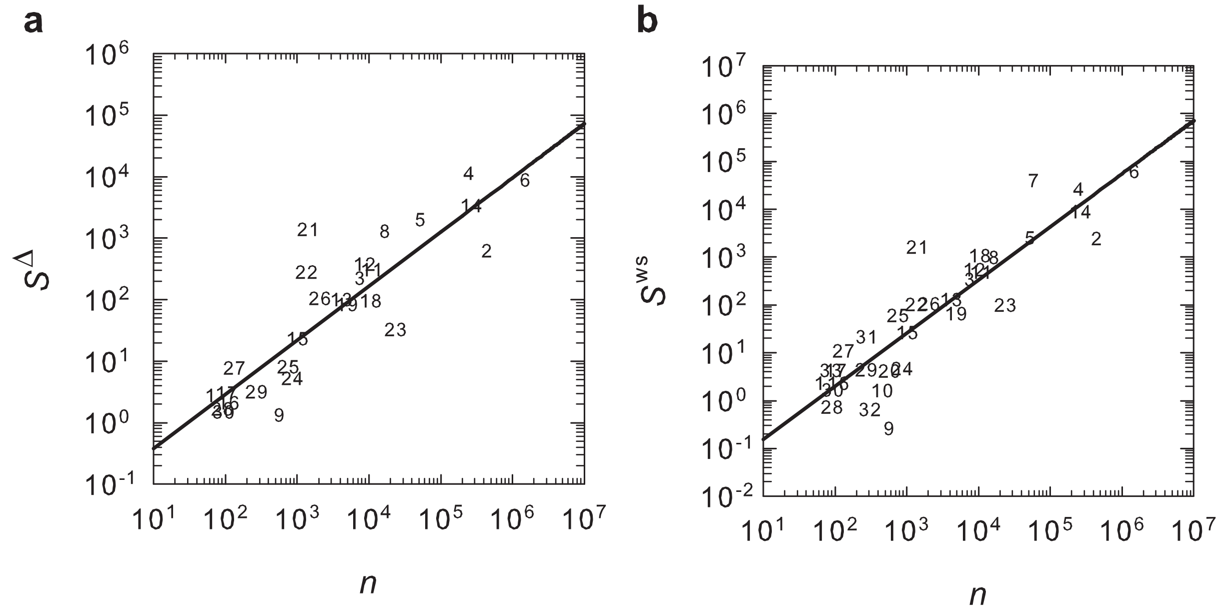

Figure ST1: Correlation of real-world system properties. Both $S^{\triangle}$ (**a**) and S^{ws} (**b**) scale linearly with network size n across real networks from all domains, and irrespective of other properties. Numbers correspond to entries in Table 1.

The few systems that were not small-world networks were defined as such according to their S^{ws} values, and not their $S^{\triangle}$ values. This illustrates the comment made in the main text that C^{ws} and $C^{\triangle}$ often considerably differ, and actually describe two different graph properties: one interpretation is that C^{ws} measures average local edge density and that $C^{\triangle}$ measures the proportion of closed loops in the network. We can see the difference clearly in Figure ST2, which shows the correlation of C^{ws} and $C^{\triangle}$ for the real-world systems in Table 1 (main text).

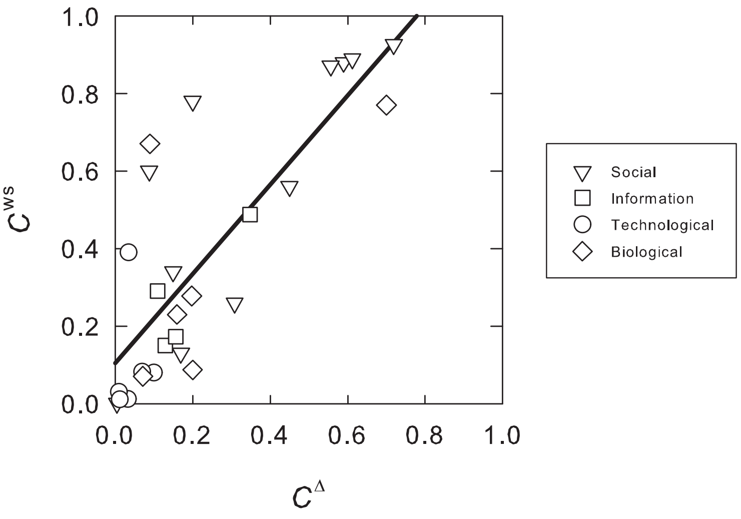

Figure ST2: Correlation of clustering coefficients for real-world systems. Linear regression shows some correlation ($r^2 = 0.65, n = 27$), but C^{ws} and $C^{\triangle}$ for some systems differ by an order of magnitude.

2 Finding maximum $S^{\triangle}$

We wanted to find out how close the particular linear model $S^{\triangle} \simeq 0.023n$ was to the theoretical maximum possible value for $S^{\triangle}$ from the Watts-Strogatz (WS) model, given the corresponding mean degree $\langle k \rangle \simeq 5$ for that data-set. To do this, we differentiated $S^{\triangle}_{ws}$ with respect to p; as $S^{\triangle}_{ws}$ has a unique maximum value, finding the value of p for which $\mathrm{d}S^{\triangle}_{ws}/\mathrm{d}p = 0$ would thus give us the theoretical maximum $S^{\triangle}$ value.

The ratios λ_{ws} and $\gamma^{\triangle}_{ws}$ can be expressed as (see main text):

$$\lambda_{ws} = \frac{n \ln(2K) f(nKp)}{K \ln(n)}, \tag{1}$$

$$\gamma^{\triangle}_{ws} = \frac{3K - 3}{8K^2 - 4K} n(1-p)^3. \tag{2}$$

If we assume that the product $nKp \gg 1$, and thus substitute the asymptotic limit $f(x) = \ln(2x)/4x$

into (1), we get the full expression

$$S_{ws}^{\triangle} = \frac{\gamma_{ws}^{\triangle}}{\lambda_{ws}} = \frac{(3K-3)n(1-p)^3 4K^2 \ln(n) p}{(8K^2-4K)\ln(2K)\ln(2nKp)}. \quad (3)$$

We want to differentiate this with respect to p, so gather all constant terms in (3)

$$S_{ws}^{\triangle} = \beta \frac{p(1-p)^3}{\ln(2nKp)}, \quad (4)$$

where

$$\beta = \frac{4K^2(3K-3)n\ln(n)}{(8K^2-4K)\ln(2K)}, \quad (5)$$

and differentiate (4) to obtain

$$\frac{\mathrm{d}S_{ws}^{\triangle}}{\mathrm{d}p} = \beta \left\{ \frac{\ln(2nKp)\left[(1-p)^3 - 3p(1-p)^2\right] - (1-p)^3}{\ln(2nKp)^2} \right\}, \quad (6)$$

We set $\mathrm{d}S_{ws}^{\triangle}/\mathrm{d}p = 0$ and re-arrange to find p. No closed form solution exists, but after some algebra we find

$$0 = (1-p)\left(1 - \frac{1}{\ln(2nKp)}\right) - 3p. \quad (7)$$

We use a standard minimisation routine — fzero from MATLAB (The MathWorks, Natick, MA), with an initial value of $p = 0.5$ — to find values for p which satisfy this equality, given $n \in [10^3, 10^4, ..., 10^{20}]$ and $K = \langle k \rangle / 2 = 2.5$. These are shown in Figure ST3a. We see that the value for p that maximises $S_{ws}^{\triangle}$ is surprisingly restricted across the whole range of n, converging on an asymptotic value of $p = 0.246$ as $n \to \infty$. If we substitute the values for n and the resulting p values into (3) we find the linear relationship $S_{ws}^{\triangle} = 0.181n$, shown in Figure ST3b. Thus, as shown in the main text (Figure 2a), the linear rate of real-world scaling does not reach the theoretical maximum.

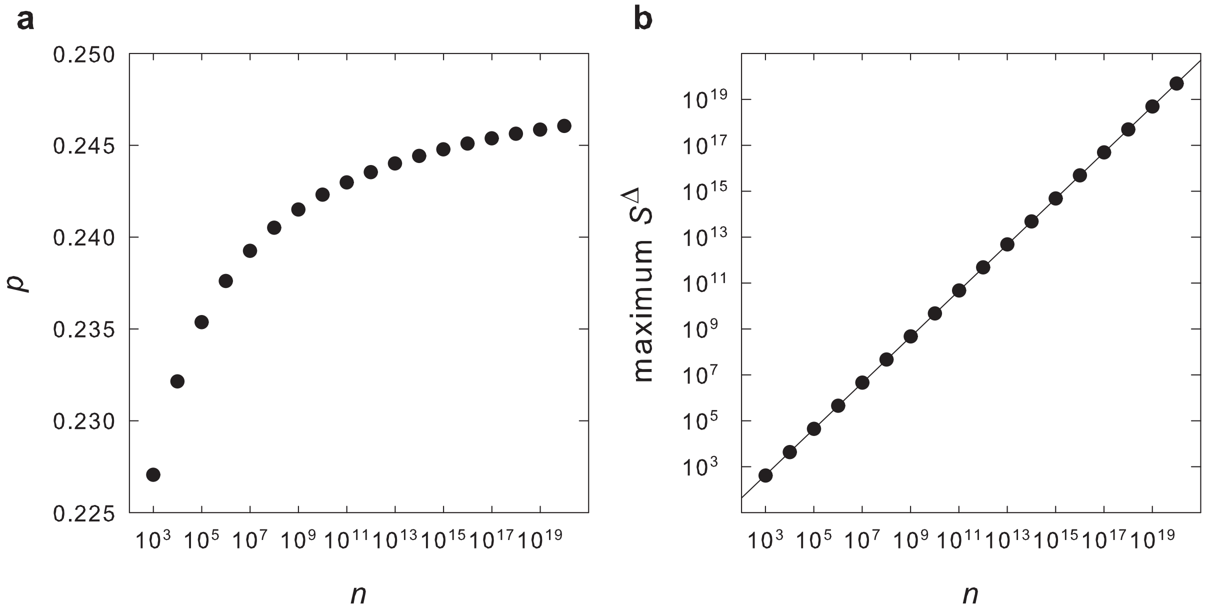

Figure ST3: Determining maximum possible $S^{\triangle}$. **a** The value for p that maximises $S^{\triangle}_{ws}$ in the WS model falls in a narrow range over many orders of magnitude of n, converging on an asymptotic value of $p = 0.246$ as $n \to \infty$. **b** The result is that maximum $S^{\triangle}_{ws}$ grows linearly with n.
